# A Systematic Review of the Effects of Urban Living on Suicidality and Self-Harm in the UK and Ireland

**DOI:** 10.1007/s11524-022-00611-z

**Published:** 2022-04-04

**Authors:** Rose-Marie Satherley, Cassie M. Hazell, Christina J. Jones, Paul Hanna

**Affiliations:** 1 School of Psychology, Department of Psychological Interventions, University of Surrey, Guildford, GU2 7XH UK; 2 School of Social Sciences and Humanities, University of Westminster, London, W1W 6UW UK

**Keywords:** Urban, Self-harm, Suicide, Deprivation, Mental health

## Abstract

We conducted a systematic review to answer the following: (a) Is there any evidence to support increased prevalence of suicidality and self-harm (i.e. self-harm or suicidality) in urban versus rural environments? (b) What aspects of the urban environment pose risk for suicidality and self-harm? Thirty-five studies met our criteria. Our findings reflect a mixed picture, but with a tendency for urban living to be associated with an increased risk of suicidality and self-harm over rural living, particularly for those living in deprived areas. Further research should focus on the clustering and additive effects of risk and protective factors for suicidality and self-harm in urban environments.

## Introduction

Over half of the world’s population live in urban environments with the figure expected to rise to 68% by 2050 [1]. Whilst this progressive urbanisation may be considered a marker of developmental progress, such a shift also presents a range of challenges including population density, concentrated areas of poverty, disconnection from natural environments, increased noise and air pollution, and social isolation [2, 3]. In conjunction with the general shift towards more urban living, we have seen an increase in the diagnosis of common mental disorders and a rising prevalence of self-harm and suicide. In 2015, the World Health Organisation identified suicide as the second leading cause of death among those aged 15–29 years, and seventh in those 30–39 years [4]. In the UK, data published by the Office of National Statistics in 2019 showed that suicide rates were increasing [5].

The similar upward trend of urbanicity and suicidality and self-harm has led researchers to consider the interrelationships between urban living and wellbeing. These findings largely highlight the detrimental effects of urban living on the population’s mental health [6]. Specifically, low socioeconomic status, social segregation, and low social capital are well-evidence risk factors for impaired wellbeing [7]. More recently, other aspects of urban living, including limited exposure to nature, the built environment, noise, and air pollution, have been associated with both depression and common mental health disorders [8, 9]. Despite research increasingly addressing the relationship between the urban environment and mental health, we know comparatively less about the role of the urban environment in suicidality and self-harm [10–12].

One review in this area identified a stark urban–rural difference in suicide rates, whereby worldwide rates of suicide were highest in urban areas [13]. However, these figures reflect a one-dimensional conceptualisation of suicide that also masks potential between-country differences. The narrow focus of analysing suicide deaths has issues around accuracy and ignores substantial complexity in this area. Rates of completed suicide are dependent on coroner reports which have inherent flaws and are likely result in an underestimation [14]. Key theoretical models in this area acknowledge the importance of suicidal ideation and intentions, and self-harm in understanding suicide risk [15, 16]. Moreover, suicide rates are likely to vary between countries, given the significant heterogeneity in both global urban and rural living standards [17]. In the UK in particular, urbanicity is a pertinent issue. In 2019, 82.9% of England’s population were living in urban areas, with future projections predicting further increases [18].

We assert a more localized and nuanced analysis of rural–urban differences in suicidality is needed. Given the growth of urbanicity in the UK and Ireland, we have focussed our review on the literature conducted in these specific localities. We have also widened the definitions of suicide used in previous reviews to include completed suicides as well as suicidal ideation and intentions, and all forms of self-harm. This systematic review aims to answer the following questions: (a) Is there any evidence to support increased prevalence of suicidality and self-harm in urban versus rural environments? (b) What specific aspects of the urban environment pose particular risk in terms of suicidality and self-harm?

## Materials and Methods

### Registration

The systematic was registered prospectively on PROSPERO, and no amendments to this protocol were made (reference: CRD42020165785).

### Search Strategy and Selection Criteria

The following databases, AMED, BNI, CINAHL, EMBASE, HBE, HMIC, MEDLINE, PsycINFO, and PubMed, were searched from inception to the end of December 2019. Searches were not updated due to the imminent coronavirus pandemic. It was thought that data from the pandemic would be atypical and therefore not representative of typical times, given the established increase in suicidality and self-harm throughout the pandemic [19–21]. Additional searches of the reference lists of articles eligible for inclusion were also conducted.

Full search terms are provided in Appendix I. These search terms were derived by reviewing the search terms used in previous systematic reviews on urban living [22] or suicide and self-harm [23].

#### Inclusion and Exclusion Criteria

Suicidality and self-harm were operationalized as any incidence of self-harm and/or suicidality, including completed or attempted suicide rates and suicide ideation.

To be eligible for inclusion, studies had to (1) present empirical data, (2) be available in English, (3) include a measure of suicide (either actual rates or ideation) and/or self-harm, (4) include a measurement and/or condition explicitly related to an aspect of urban living, (5) report data from the UK and Ireland, and (6) be published in a peer-reviewed journal. We classified aspects of urban living as features of the built environment (e.g. buildings, transport), and the environmental elements of urban living (e.g. access to green space, noise, pollution). Social aspects of the urban environment, namely socioeconomic status, social fragmentation, and characteristics of local communities, were also considered important features of the urban environment.

The following were excluded: (1) protocol, theoretical, or position papers; (2) studies focusing on assisted suicide; (3) studies measuring suicide or self-harm stigma only; (4) studies focussing on rural living only (without an urban comparison); (5) those focussing on murder/homicide preceding suicide; (6) those focussing on substance abuse or overdose (where intent cannot be established); (7) those including a measurement/condition related to geography and/or living but this is not explicitly tied to urbanicity; (8) reports data from the UK that could not be separated from multi-national data; or (9) reports suicide/self-harm rates in the context of persons with a specific condition (e.g. people with a particular physical or psychological difficulty). Studies were not excluded based on the methodology used.

### Screening Procedure

**Fig. 1:**
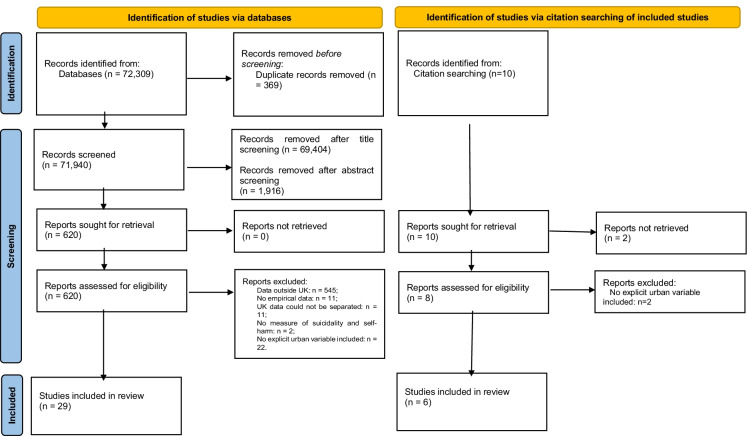
PRISMA diagram

First, one author screened studies by their title and removed duplicates, and after which, two authors separately screened papers by their abstract, and finally by the full paper. The screening procedure and papers removed at each stage of the screening process are outlined (Fig. 1).

### Data Extraction and Quality Assessment

Studies were independently reviewed by the research team, who extracted information relating to (1) author and year of publication; (2) study population, including sociodemographic details or data resource where relevant; (3) definition of urban–rural categorisation; and (4) measures relating to urbanicity and suicidality and self-harm.

Quality assessment was completed independently by three authors, using the National Institute of Health’s tool for cross-sectional research, which requires reviewers to rate 14 statements about research quality [24]. Each statement is rated on a three-point scale, good, fair, or poor. The overall quality rating of each study focusses on 14 key concepts (e.g. study population, sample size, confounding variables) and how this impacts study validity. Statements are not used to develop an overall quality score but used to inform an interpretation of overall study quality. One-third of included studies were quality assessed by more than one author; an interrater reliability analysis using the Kappa statistic was performed to determine consistency among the research team.

### Data Synthesis

Extracted data was entered into evidence tables showing study characteristics and results. Variation in definitions for urbanicity across studies made the data unsuitable for meta-analysis. Analysis across studies was completed, comparing characteristics, methods, and findings, and a narrative synthesis of findings was applied to summarize the strength of evidence. Given the financial, social, and environmental changes in urban environments, the time period in which data was collected is highlighted. As there is no established time period in which these changes to urban environments took place, we used a descriptive assessment of time periods where appropriate.

For the first research question, evidence to support increased prevalence of suicidality and self-harm in urban versus rural environments, unadjusted and adjusted comparisons were extracted from those studies that compared rates of suicidality and self-harm across rural and urban environments. For the second research question, aspects of the urban environment that pose particular risk in terms of suicidality and self-harm, only those studies that specifically reported correlates of suicidality and self-harm in urban environments were included. Where risk factors for suicidality and self-harm were associated with the living environment in general, but not urban living specifically, data was not extracted.

## Results

We identified 35 relevant studies that met the inclusion criteria in the search (Fig. 1). An overview of the results from each study is provided (Table 1).

**Table 1:** Overview of included studies and study characteristics

Author (date)	Date source* (years)	Sample size (gender, age, region)	Definition of urban environment	Suicidality and self-harm indicator	Aspects of urban environment related to suicidality and self-harm	Prevalence of suicidality and self-harm in urban vs rural environments
Capstick (1960) [25]	Coroners Records of Suicides (1951–1955)	881 events/persons (males and females, resident in Wales)	Population density	Deaths recorded as suicide		Rates of suicide were associated with greater urbanicity, but rates ranged from 6.16–28.35 per 100,000 population Elevated suicide rates were also evident for men living in sparsely populated rural environments
McCulloch, Philip, Carstairs (1967) [26]	Official Register of Death by Suicide in the City of Edinburgh (1963–1965)	216 events/persons (48.1% male, 15–94 years, resident in Edinburgh)	Edinburgh, Capital City of Scotland	Deaths recorded as suicide	↑* Area level overcrowding, tenement housing, owner-occupied housing ↑* Area level school absences, children in care, juvenile delinquency	
Obafunwa, Busuttil (1994) [27]	Record of Sudden or Violent and Unexplained Deaths for Lothian and Borders Region (1987–1991)	400 events/persons (69.3% male, resident in Lothian and Borders Region of Scotland)	Not defined	Deaths recorded as suicide		
Lyster, Youssef (1995) [28]	Referrals to Louth Co. Hospital for Psychiatric Assessment following Suicide Attempt (1992)	95 events/persons (40% male, resident in Dundalk, Republic of Ireland)	Not defined	Referrals for psychiatric assessment following suicide attempt		
Congdon (1996) [29]	Not Specified (1990–1992)	Males and females, resident in Greater London	London, Capital City of England, divided into inner-city areas and suburban areas	Suicide attempt or death recorded as suicide		
Saunderson, Langford (1996) [30]	Office of Population Censuses and Surveys (1989–1992)	Males and females, 15–64 years, resident in England or Wales, excluding the City of London or Isles of Scilly	Not defined	Deaths recorded as suicide		
Saunderson, Haynes, Langford (1998) [31]	Office for National Statistics (1989–1992)	5,782 events/persons (50.9% male, resident in England and Wales)	Population density	Deaths recorded as suicide or undetermined cause		
Kennedy, Iveson, Hill (1999) [32]	Coroners Records of Suicides (1993–1996)	2,734 events/persons (resident in London)	Population density	Deaths recorded as suicide	↑* Socioeconomic deprivation ↑* Area level violence and homicide ↔ ethnicity	Suicide occurred more frequently in highly populated areas, with an exponential increase, with steep rises in rates above a population density of 50 per hectare Socioeconomic deprivation accounted for most, if not all local variation in suicide
Gunnell, Shepherd, Evans (2000) [33]	Survey across three emergency departments (1972–1973) Bristol Deliberate Self Harm Register (1995–1996)	3,576 persons (males and females, > 15 years resident in Bristol. England)	Bristol, City in England	Deliberate self-harm	↑* Socioeconomic deprivation ↑* Social fragmentation	Rates of self-harm increased between 1995–1996 and 1972–1973, alongside increases in socioeconomic deprivation
Connolly, Lester (2001) [34]	National Population Census (1988–1984)	Resident in Irish Counties	Composite score (gender, % illegitimate births, female labor force, % population urban)	Deaths recorded as suicide		During 1978–1986, there was no association between living environment and suicide. In 1988–1994, suicide rates were associated with greater urbanicity
Kelleher et al. (2002) [35]	National Population Census (1976–1994)	Males and females, resident in Ireland	Place of residence	Deaths recorded as suicide or undetermined cause		Prior to 1980, suicide rates were greater for females in urban environments. Post 1980, overall male suicide rates increased by 50% but no change in urban rates
Middleton et al. (2003) [36]	Office for National Statistics (1981–1998)	Males and females, 15–44 years, resident in England or Wales	Population density and population potential	Deaths recorded a suicide or undetermined cause		In the 1980s, suicide rates were higher in urban environments but by the 1990s, these urban–rural differences had narrowed. Over time, there was an increase suicide rates in rural areas, this effect was most marked in females (15–24-year-olds) After adjustment for socioeconomic deprivation, the strength of risk associated with rural environments did not change. Areas characterized by markers of low social fragmentation had the highest rates of suicide
Middleton et al. (2004) [37]	Office of National Statistics (1991–1993)	16,215 events/persons (male and female, > 15 years, resident in England or Wales)	Population density	Deaths recorded as suicide or undetermined cause		Suicide rates were greatest in the most urban and most rural environments, with notable patterns of male suicide in sparsely populated areas After adjustment for socioeconomic deprivation, suicidality and self-harm were no longer associated with urbanicity
Stark et al. (2004) [38]	General Register Office for Scotland (1981–1999)	14,502 events/persons (71.5% male, resident in Scotland)	Population density	Death recorded as suicide		Male suicide rates were elevated in the rural Western Isles, and in urban areas like Greater Glasgow, female suicide rates were higher in Greater Glasgow only
Levin, Leyland (2005) [39]	General Registrar Office for Scotland (1981–1999)	(resident in Scotland)	Population density	Deaths recorded as suicide, self-inflicted injury or undetermined cause	↑* Socioeconomic deprivation	Rates of suicide were positively associated with living in a rural environment. Suicide rates were high for men living in rural areas After adjustments for socioeconomic deprivation, rurality was no longer significantly associated with suicide
Middleton, Sterne, Gunnell (2006) [40]	Office for National Statistics (1988–1994)	1221 events/persons (males, 15–44 years, resident in England or Wales)	Population density	Deaths recorded as suicide or undetermined cause	↑* Socioeconomic deprivation	High concentrations of suicide were found in both inner-city areas and remote, or coastal rural areas The higher rates of suicide were found in inner city areas were largely explained by the socioeconomic characteristics of these areas. Socioeconomic deprivation could not fully explain the clusters of self-harm evident in remote coastal areas
Stark et al. (2007) [41]	General Registrar Office for Scotland (1981–1999)	(Males and Females > 15 years, resident in Scotland)	Population density	Death recorded as suicide or undetermined cause	↑* Socioeconomic deprivation	Overall, rates of suicide were positively associated with population density across all age groups, other than young women, but local area variation was high Rates of suicide were greatest in the most urban and the most rural areas
Rezaeian, Dunn, Leger, Appleby (2007) [42]	National Confidential Inquiry into Suicide and Homicide by People with Mental Illness (1996–1998)	2,190 events/persons (males and females, resident in London)	London, Capitol City of England	Deaths recorded as suicide or probable suicide	↑* Socioeconomic deprivation	Suicide rates were greater in inner-city areas when compared to outer-city areas
Corcoran, Arensman, Perry (2007) [43]	Irish National Registry of Deliberate Self Harm (2002–2004)	25,797 persons, 32,777 events (males and females, resident in Republic of Ireland)	State definition of urban vs. rural districts	Hospital emergency department presentations for deliberate self-harm	↑* Socioeconomic deprivation ↑* Social fragmentation	Overall, rates of self-harm were higher in urban (vs rural) environments. Variation was apparent, with the City of Dublin having lower rates of self-harm than other Irish cities, despite being the most urbanized After adjusting for fragmentation and deprivation, a small, but significant residual relationship remained between urban environment and self-harm. Deprivation was the strongest area-level predictor of self-harm
Mitchell, Popham (2008) [44]	National Office for Statistics (2001–2005)	366,348 events (males and females, resident in England)	Quantity of green space	Deaths recorded as intentional self-harm		No differences in rates of suicidality and self-harm between urban–rural environments No interactions between socioeconomic deprivation and environment were found in relation to suicidality and self-harm and living environment
O’Reilly, Rosato, Connolly, Cardwell (2008) [45]	Northern Ireland Statistics and Research Agency (2001–2006)	566 events/persons (Males and females, 16–74 years, resident in Northern Ireland)	Population density	Death recorded as suicide		No significant relationship was found between population density and suicide risk Indicators of socioeconomic disadvantages were strongly related to suicide risk, with higher rates in socially fragmented and deprived areas, with population density no longer apparent in the fully adjusted model
Sarma, Kola (2010) [46]	Central Statistics Office of Ireland (1980–2005)	9,674 events/persons (males and females, resident in Ireland)	Place of residence	Death recorded as suicide		Those completing suicide by hanging were more likely to be rural dwelling
Cooper et al. (2010) [47]	Emergency department records across three general hospitals (2001–2006)	14,997 persons (males and females, 16–64 years seeking treatment in Oxford, Manchester, or Derby)	Cities of Oxford, Manchester, and Derby	Self-harm presentations to emergency departments	↑* Young, black females	
Harriss, Hawton (2011) [48]	Oxford Monitoring System for Attempted Suicide (2001–2005)	4054 persons, 6833 events (40.6% male, > 15 years, residence in Oxford, England)	Population density and wider surroundings	Hospital presentations for deliberate self-harm	↑* Socioeconomic deprivation ↑* Social fragmentation ↑* “Non-white ethnic origins”	Self-harm rates were greater for those living in urban environments Higher levels of both deprivation and social fragmentation partially explained but could not fully explain clusters in urban environments
Gartner, Farewell, Roach, Dunstan (2011) [49]	Office for National Statistics (2002–2004)	4,780 events/persons (Males and Females, resident in England or Wales)	Population density	Deaths recorded as suicide	↑* Socioeconomic deprivation	Prior to adjustment, suicide was more common for those living in urban areas After adjustment for socioeconomic deprivation, direction of relationships changed, with rates of suicide appearing greater for men living in rural areas. Not adjusting for deprivation appeared to mask the increase in male suicide rates in rural areas. Socioeconomic deprivation explained the differences across urban–rural environment in females. Choice of deprivation measure did not alter the analyses
Congdon (2011) [50]	Not Specified (1992–2007)	Males and females, > 15 years, resident in East and South East England	Population density	Self-harm admissions and deaths recorded as suicide		Instances of self-harm were greater in urban environments, even after accounting for socioeconomic deprivation Gender effects were apparent. For females, social fragmentation had influenced suicide risk, but for males, socioeconomic deprivation was the strongest predictor. Effects of urbanicity remained, despite controlling for socioeconomic deprivation
Arensman et al. (2014) [51]	Irish National Registry of Deliberate Self Harm (2003–2010)	55,288 persons, 87,085 events (males and females, resident in Republic of Ireland)	Place of residence	Hospital emergency department presentations for self-harm		Self-cutting was more common in urban environments When including gender, age, living circumstances, and clinical factors in multinomial logistic regression models, an independent positive association remained between self-cutting and urban residence
O’Farrell, Corcoran, Perry (2015) [52]	Irish National Registry of Deliberate Self Harm (2009–2011)	26,379 persons (males and females, 15–64 years, resident in Republic of Ireland)	Population density	Hospital treated self-harm patients	↑* Socioeconomic deprivation ↑* Social fragmentation	Instances of self-harm were positively associated with greater urbanicity When adjusting for area level variables, suicidality and self-harm remained greater for those living closer to hospitals
Bixby et al. (2015) [53]	Office of National Statistics (2002–2009)	5,222 events/persons (79.9% males, 15–64 years, resident in England)	Proportion of green space	Death recorded as suicide	↔ Urban green space	
O’Farrell, Corcoran, Perry (2016) [54]	Irish Central Statistics Office (2009–2011)	1,654 events/persons (Male and females > 15 years, resident in Republic of Ireland)	Population density	Deaths recorded as suicide or undetermined cause		Overall, suicide was more common in rural environment, except for males (40–64 years) where rurality was associated with a decreased risk of suicide Relationships between population density and suicide remained almost unchanged after adjustment for deprivation and fragmentation. Socioeconomic deprivation was the strongest predictor of area-level suicide rates
Kar (2016) [55]	Coroners Records of Suicides (2004–2011)	146 events/persons (74.7% male, 16–88 years, resident in Wolverhampton)	Wolverhampton, City in England	Deaths recorded as suicide		Instances of suicide were greater in inner city wards compared to outer wards
Grigoroglou et al. (2018) [56]	Office for National Statistics (2006–2014)	38,511 events (Males and Females, > 20 years, resident in England)	Not defined	Deaths recorded as intentional self-harm, injury/poisoning or undetermined intent and sequelae of intentional self-harm		Suicidality and self-harm was positively associated with greater rurality In binomial regression models, elevated suicide was associated with greater social fragmentation and deprivation, but the strongest predictor was rurality. No associations between suicidality and self-harm and quality of mental health services in the local area
Polling et al. (2019) [57]	Clinical Records Interactive Search System, linked to Hospital Episode Statistics (2007–2016)	8,327 events/persons (39.4% male, > 15 years assessment in individual’s resident across four London boroughs, England)	Population density, proximity to the city center and percentage green space	Hospital admissions for self-harm	↑* Socioeconomic deprivation	Rates of hospital admissions for self-harm were less for those living close to the city center, compared to those living further away Rates of self-harm within the city were not explained by area-level socioeconomic deprivation and some deprived inner-city areas had paradoxically low rates After adjustment for deprivation, hospital of admission and social fragmentation, greenspace, population density and ethnicity were not associated with self-harm rates
Congdon (2019) [58]	Office for National Statistics (2012–2016)	23,517 events/persons (76.3% male, resident in England)	Based on 2011 Census	Deaths recorded as suicide		Rates of suicide were comparable across urban and rural environments After adjustment for fragmentation and deprivation, the effects of increasing rurality on suicidality and self-harm remained but were less strong as that of deprivation and fragmentation. Deprivation was a stronger predictor for male suicide, and fragmentation was a stronger predictor for females
Griffin et al. (2019) [59]	Northern Ireland Self Harm Registry (2013–2015)	22,307 events, 14,477 persons (50% Male, 16–64 years, resident in Northern Ireland)	Population density	Self-harm presentations to emergency departments		Rates of self-harm were more than three times higher in the most densely populated areas (IRR = 3.47, 95% CI = 3.08–3.92) After adjustments for socioeconomic deprivation and fragmentation, moderate associations between self-harm rates and living in urban environments remained but varied according to gender. For males, rates were 67% higher in the most densely populated areas

*Note:* *ordered by time period, from earliest start date to latest

### Study Characteristics

Sample sizes ranged between 95 and 366,348 persons; these were individual participants, as opposed to events of suicidality and self-harm (one participant may experience more than one event of self-harm or suicide attempt). Eight studies did not record the total number of persons [29, 31, 34, 35, 37, 39, 41, 50]. Most studies were conducted in England only (*n* = 13), with eight in the Republic of Ireland, six in England and Wales, five in Scotland, two in Northern Ireland, and one in Wales only.

### Definitions of Urban–Rural Environments

Definition and categorisation of urban areas varied greatly across included studies. Population density was used as a proxy indicator for levels of urbanicity across most studies (40%), three inferred population density by participants home residence, two used the proportion of green space, and two used a combination of population density, proximity to the city center, and the proportion of green space. Four studies did not provide a definition and two used “state-defined” classifications but did not expand further. Five studies were completed across urban cities (Edinburgh, London, Wolverhampton, Bristol, with one study focussing on three cities, Oxford, Derby, and Manchester) [26, 33, 42, 47, 55], and one divided the city of London into inner and outer city areas [29]. One based the definition of urban–rural environment on a combination or variables derived from factor analysis [34].

#### Measurement of Suicidality and Self-Harm

Suicidality and self-harm indicators varied, with 12 (34%) studies measuring this as intentional self-harm, 20 (57%) as suicide, and 1 as suicide attempt. One study used a combination of intentional self-harm and death recorded as suicide [50], and another as a combination of suicide attempt and death recorded as suicide [29].

Instances of suicide were assessed via publicly held records (e.g. coroner’s reports and Census data); one assessed suicide attempt through referrals for psychiatric assessment post suicide attempt [28]. Measurement of self-harm was assessed via local self-harm registries based on hospital presentations for self-harm across all studies except Gunnell et al. [33] who initially assessed self-harm through a survey across three emergency departments in the 1970s, and then followed this up in the 1990s with the local self-harm registry.

### Quality Assessment

Inter-rater agreement for quality assessment for included studies across reviewers was moderate (Kappa statistic = 0.74–0.79); nineteen of the included studies were determined to be of good quality (54%). Notably, ten studies did not report the age or gender of their population. Fourteen studies did not include any key confounding variables to adjust for the impact of relationships between urban–rural environment on suicidality and self-harm. Confounding variables that were adjusted for included gender or age, as well as area-level socioeconomic deprivation or social fragmentation, both of which were defined by established measures obtained from Census data. Only five studies examined different levels of urbanicity in relation to suicidality and self-harm [43, 44, 49, 52, 57].

### Evidence to Support Increased Prevalence of Suicidality and Self-Harm in Urban Versus Rural Environments

Twenty-eight studies provided unadjusted rural–urban comparisons for suicidality and self-harm. The majority (54%; 6 assessing self-harm, 9 assessing suicide) reported significant associations between greater urbanicity and increased rates of suicidality and self-harm. Rates of suicide ranged from 6.16–28.35 per 100,000 in urban areas, and 1.78–10.5 per 100,000 in rural areas [25, 27, 32]; one study reported rates of self-harm to be more than three times higher in more densely populated areas (IRR = 3.47, 95% CI = 3.08–3.92) [59].

In contrast, six studies (21%; 5 assessing suicide, 1 assessing self-harm) reported positive association between suicidality and self-harm and greater rurality. Four studies (14%; 3 assessing suicide, 1 assessing self-harm) reported no difference in rates of suicidality and self-harm across urban–rural environments [28, 44, 45, 58], and three (11%; all assessing suicide) reported clusters of suicidality and self-harm in both urban and rural areas, demonstrating apparent U-shaped associations [30, 37, 38].

Although reporting associations with suicidality and self-harm across urban–rural environments, several authors noted challenges with these interpretations, given inconsistencies within rural and urban environments [25, 31, 41, 43]. For example, overall, Corcoran, Arensman, and Perry reported higher rates of self-harm in urban (vs rural) environments, but reported lower rates of self-harm in Dublin, the capital city of Ireland, than other Irish cities, despite being the most urbanized city [43].

An additional four studies assessed changes in rates of suicidality and self-harm across urban–rural environments between 1972 and 1996; again, results were inconclusive [33–36]. Over time, two studies reported an increase of suicidality and self-harm rates in urban environments [24, 33, 34]. However, two further studies reported a narrowing of urban–rural rates of suicidality and self-harm over time, resulting from an increase in rates of suicidality and self-harm in rural environments over more recent years [35, 36].

#### Adjusted Associations

Of the fifteen studies that reported greater rates of suicidality and self-harm in urban (vs rural) areas, six (40%) adjusted for potentially confounding variables within the urban environment. Two adjusted for area level social fragmentation and socioeconomic deprivation [43, 59]; small but significant relationships remained between urbanicity and self-harm after adjustment in both these studies. Five studies adjusted for area level socioeconomic deprivation only [32, 37, 40, 43, 49]; this socioeconomic deprivation largely explained higher rates of suicide in urban areas (3/5 studies). Corcoran et al. [43] reported instances of self-harm were still greater in urban environments after accounting for area level socioeconomic deprivation. Gartner et al. [49] found that adjustment for deprivation changed the direction of relationships; although deprivation explained urban–rural differences in female suicide, after adjustment, suicide appeared greater for men in rural areas. The authors concluded that not adjusting for deprivation appeared to mask the increase in male suicides in rural areas. One additional study adjusted for individual living circumstances and clinical factors [51], after adjustment, an independent positive association remained between self-harm and urban residence.

For those that reported greater rates of suicidality and self-harm in rural (vs urban) environments, after adjustment for area level, socioeconomic deprivation and/or fragmentation did not change the strength of risk of suicide associated with rural environments in 3/5 studies [36, 54, 56]. In contrast, two studies reported that population density was not associated with suicide [39] or self-harm [57] when adjusting for area level socioeconomic deprivation.

### Aspects of the Urban Environment that Pose Particular Risk in Terms of Suicidality and Self-Harm

Twenty (57%) of the included studies assessed at least one aspect of the urban environment in relation to the risk of suicidality and self-harm. Aspects of the urban environment assessed included ethnic diversity of the area, area level socioeconomic deprivation, social fragmentation, crime, and features of the built environment.

#### Features of the Built Environment

Surprisingly, only two studies assessed the role of environmental features. Bixby et al. [53] reported no association between the presence of urban green space and suicide rates across England, whereas McCulloch et al. [26] reported greater suicide rates in Scottish urban areas with greater overcrowding and tenement housing.

#### Urban Crime

Area-level crime or juvenile delinquency [26, 32] was associated with increased rates of suicide in urban areas. In a review of coroner’s records within the city of Bristol, areas with high homicide and violence were associated with increased instances of suicide [32].

#### Ethnic Diversity

Three studies assessed ethnicity as a risk factor for self-harm (*n* = 2) and for suicide (*n* = 1). Self-harm in the city of Oxford, was more likely to be completed by “non-white ethnic” individuals, 48 and across the cities of Oxford, Manchester and Derby self-harm was more common in young black females [47]. In comparison, there was no association between ethnicity and suicide in London [32].

#### Socioeconomic Deprivation

Area-level deprivation was assessed using a variety of measures; the most common was the Townsend Index (*n* = 4). Twelve studies identified area-level indicators of socioeconomic deprivation as a risk factor for suicidality and self-harm in urban environments, irrespective of the location, date of data collection, or suicidality and self-harm type. However, local variations in suicidality and self-harm were apparent across urban areas; some urban areas had lower suicide rates than expected, given their high socioeconomic deprivation scores [32, 36, 40].

The effect of urban environment varied by gender; two studies completed by the same author, reported on the gendered effects of socioeconomic deprivation. For males, suicide attempts and completed suicide in urban environments were most strongly influenced by socioeconomic factors [29, 50].

#### Social Fragmentation

All four studies assessing area-level social fragmentation, typically assessed via the Congdon index [29], concluded that great area-level social fragmentation increased the risk of suicidality and self-harm in urban areas [33, 43, 48, 52]. However, on further examination, for young adults in Dublin, areas of higher area-level social fragmentation were associated with lower rates of self-harm [43]. The authors suggest this finding as an artefact of the measures of social fragmentation, which may be limited in inner city areas characterized by a young, unmarried population, who more often live alone. Again, gendered effects were apparent. Females attempting or completing suicide in urban settings appeared most influenced by social factors, in contrast to males who were mist influenced by socioeconomic factors [29, 50].

## Discussion

This systematic review identified 35 studies reporting on suicidality and self-harm in urban environments across the UK, prior to the coronavirus pandemic. We identified varied and often contradictory outcomes across studies, which were often limited by definitions of urbanicity, and measurement of suicidality and self-harm. Across most included studies, living in an urban environment was associated with an increased risk of suicidality and self-harm, compared to rural living, but findings were inconsistent.

Area-level socioeconomic deprivation and social fragmentation appeared to increase the risk of suicidality and self-harm for those living in urban environments. This result is not surprising, as both have been highlighted as key risk factors for suicidality and self-harm across several academic, clinical, and policy reports [7, 60]. However, relationships between deprivation, fragmentation, and suicidality and self-harm were not as consistent as we would perhaps expect, with variation apparent within urban environments and evidence for gendered effects of urban living on suicidality and self-harm; males in urban areas appeared more influenced by socioeconomic factors, whereas females in rural areas appeared more influenced by social fragmentation [29, 50]. These variations may result from the way in which area-level fragmentation and deprivation is assessed. For example, Congdon suggests that indicators used to measure socially fragmented communities may not measure fragmentation, but rather younger communities with young professionals or students, especially within urban areas [29]. Similarly, assessment of socioeconomic deprivation relies on Census data which is collected every 10 years. However, urban environments are susceptible to change within short periods of time, now so, more than ever before, with the coronavirus pandemic, which Census data may fail to capture [61].

Community or environmental factors were rarely assessed in relation to suicidality and self-harm within urban environments. Notably, only one study explored the role of green space, reporting no association with suicide rates across England [53]. This is surprising, given well-established theoretical models, which point to the importance of considering the resources and environmental characteristics of urban communities that might protect against impaired mental wellbeing [62]. Across the UK and Ireland, urban areas have undergone increasing gentrification, with greater investments in housing, and the introduction of resources and services [63]. Aspects of the local environment, including community support, availability of public transport, and green space, can act as protective factors providing individuals with resources to cope with stressors [64, 65]. Without knowing more about how multiple factors interact to influence suicidality and self-harm in urban environments, it is impossible to develop interventions that address this real-world complexity.

Those living in urban environments may be disadvantaged on many levels, experiencing increased crime, social fragmentation, socioeconomic deprivation, poorer quality housing, and/or limited access to green space [66]. It is likely the cumulative stress of these factors, in combination with protective factors, which interact to impair wellbeing. To further understand the interactions between suicidality and self-harm within urban environments, greater insight on the interrelationships between social context, environment, and suicidality and self-harm is required. As summarized by Curtis et al. [67], individual factors, familial attributes, characteristics of the local community and the wider national or regional context are all likely to interact to influence wellbeing. The low rates of suicidality and self-harm in urban environments described across two studies included within this review [43, 57] highlight the need to consider the context of urban environments, and the ways in which suicidality and self-harm is experienced in different urban settings and across different populations [68].

### Future Directions

This review highlights important gaps in the design and evaluation of research that can help answer these questions. We suggest that research, policy, and practice need to go beyond the urban–rural division, focussing on the characteristics of local communities, and how interactions with local environments, spaces, and communities modify risk factors for suicidality and self-harm. The UK government’s focus on preventative and integrated approaches to care at the local level, which coordinate health and social services to meet the needs of the local community, provides a promising basis for further work [69]. In line with these frameworks, high-quality, longitudinal analyses of routinely collected data may be beneficial in exploring how these variables cluster and interact, whilst qualitative approaches have the potential to help refine population and exposures for these analyses and help identify key experiences that help individuals overcome adversity within urban environments.

From a theoretical perspective, there is a need to explore the complexity of the urban environment and its relationship with suicidality and self-harm. We are now starting to see intersectional approaches applied to our understanding of urban environments and suicidality and self-harm [70, 71]. Intersectional approaches move us beyond considering single social determinants, such as socioeconomic deprivation, instead, considering these in combination with social processes and environmental influences (e.g. social support, employment, green space). An intersectional framework may provide more precise identification and understanding of suicidality and self-harm in urban areas, prioritising the voice of those most affected by these issues.

### Strengths and Limitations

This is the first systematic review to assess the implications of urban living on suicidality and self-harm in the UK and Ireland and includes a comprehensive variety of studies conducted over time. We have been able to draw conclusions regarding urban–rural differences in suicide and self-harm risk based on 35 studies. Our conclusions regarding specific urban-related risk factors however are based on a smaller sub-set of studies and should be interpreted in light of this limitation.

Despite no restrictions on methodology, most studies included in this review were cross-sectional in nature, meaning no conclusions can be made about the causal effect of urban living on suicidality and self-harm. We acknowledge that the detailed nature of our inclusion criteria may have prevented the inclusion of qualitative studies but highlight that no qualitative or mixed method studies were included in the identified eligible studies.

To be eligible for inclusion, articles were required to assess aspects of the urban environment and suicidality and self-harm, definitions for which varied across included studies. Urban environment was largely defined via population density, although varied widely across studies. In addition, suicidality and self-harm was largely assessed via publicly held records (i.e. coroner reports). As such, the rates of suicide and deliberate self-harm reported here may be an underestimation due to the high burden of proof required to declare a death a suicide [72], and hospitals’ poor recording of non-admitted self-harm cases [73]. The results presented here should be interpreted with a clear understanding of the time in which each study collected data, given the changes in urban living over recent decades, as well as each studies definition of the urban environment. To aid interpretation, we present descriptions of the time periods in which data was collected, where relevant, and include information on this and definitions of the urban environment within our tables.

## Conclusions

There is a need for high-quality, theoretically informed research to further understand and inform preventive, local interventions to address suicidality and self-harm in urban environments across the UK and Ireland. Drawing on our findings, we highlight the limitations of urban–rural distinctions in an increasingly complex world, prioritising a focus on the relationships between urban living, protective and risk factors, as well as individual experience.

## Data Availability

The data used in this study is available directly from the papers included in this review.

## Appendix

Full list of search terms.

**Table:**

Free text words	(“suicid*” OR “overdos*” OR “self?harm*” OR “self?injur*” OR “self?cut*” OR “self?destruct*” OR “auto?mutilat*” OR “auto?destruct*” OR “self?inflict*” OR “self?poison*” OR “self?mutilat*”) AND (“Moderni?ation” OR “urban*” OR “rural*” OR “open space*” OR “park*” OR “green” OR “wood*” OR “forest*” OR “garden*” OR “environment*” OR “communit*” OR “grow*” OR “city” OR “civili?at*” OR “neighbo?rhood” OR “geography” OR “public space” OR “natur*” OR “landscape” OR “tree*”)
MeSH	
Field	Title; Abstract
Limits	None
